# Blueberries improve biomarkers of cardiometabolic function in participants with metabolic syndrome—results from a 6-month, double-blind, randomized controlled trial

**DOI:** 10.1093/ajcn/nqy380

**Published:** 2019-05-28

**Authors:** Peter J Curtis, Vera van der Velpen, Lindsey Berends, Amy Jennings, Martin Feelisch, A Margot Umpleby, Mark Evans, Bernadette O Fernandez, Mia S Meiss, Magdalena Minnion, John Potter, Anne-Marie Minihane, Colin D Kay, Eric B Rimm, Aedín Cassidy

**Affiliations:** 1 Department of Nutrition & Preventive Medicine, Norwich Medical School, University of East Anglia, Norwich, United Kingdom; 2 Clinical and Experimental Sciences, Faculty of Medicine, University of Southampton and University Hospital Southampton NHS Foundation Trust, Southampton, United Kingdom; 3 Department of Nutritional Sciences, University of Surrey, Guildford, United Kingdom; 4 Wellcome Trust-MRC Institute of Metabolic Science, University of Cambridge, Cambridge, United Kingdom; 5 Departments of Epidemiology & Nutrition, Harvard TH Chan School of Public Health, and Channing Division of Network Medicine, Brigham and Women's Hospital and Harvard Medical School, Boston, MA

**Keywords:** metabolic syndrome, blueberry anthocyanins, flavonoids, cardiovascular disease risk, anthocyanin-derived phenolic acid metabolites

## Abstract

**Background:**

Anthocyanin-rich blueberry intake is associated with reduced type 2 diabetes and cardiovascular disease (CVD) risk in prospective studies, although long-term randomized controlled trials (RCTs) have not been conducted in at-risk populations.

**Objective:**

In the longest-duration RCT to date, we examined the effect of 6-mo blueberry intake on insulin resistance and cardiometabolic function in metabolic syndrome.

**Methods:**

A double-blind, parallel RCT (*n* = 115; age 63 ± 7 y; 68% male; body mass index 31.2 ± 3.0 kg/m^2^) was conducted, which fed 2 dietarily achievable blueberry intakes [equivalent to 1/2 and 1 cup/d (75/150 g)] compared with matched placebo. Insulin resistance was assessed via the homeostasis model assessment of insulin resistance (primary endpoint) and confirmed by [6-6-^2^H_2_]-glucose-labeled, 2-step hyperinsulinemic clamp (*n* = 20). Clinically relevant cardiometabolic endpoints [including flow-mediated dilatation, augmentation index, lipoprotein status (by nuclear magnetic resonance spectroscopy), and nitric oxide (NO)-related metabolite assay] and anthocyanin metabolism were assessed.

**Results:**

A daily intake of 1 cup of blueberries improved endothelial function (flow-mediated dilatation: +1.45%; 95% CI: 0.83%, 2.1%; *P* = 0.003), systemic arterial stiffness (augmentation index: –2.24%; 95% CI: –3.97%, –0.61%; *P* = 0.04) and attenuated cyclic guanosine monophosphate concentrations. In statin nonusers (*n* = 71), elevated high-density lipoprotein cholesterol (+0.08 mmol/L; *P* = 0.03), high-density lipoprotein particle density (+0.48*n*, ×10^–6^; *P* = 0.002) and apolipoprotein A-I (+0.05 g/L; *P* = 0.01) concentrations were observed following the 1-cup/d intervention. Treatment compliance was 94.1% (wrapper returns) and total concentrations of anthocyanin-derived phenolic acid metabolites significantly increased, dose-dependently, in serum and 24-h urine (*P* < 0.01 and *P* < 0.001, respectively). Insulin resistance, pulse wave velocity, blood pressure, NO, and overall plasma thiol status were unaffected. Likewise, a half cup per day had no effect on any biomarkers.

**Conclusions:**

Despite insulin resistance remaining unchanged we show, to our knowledge, the first sustained improvements in vascular function, lipid status, and underlying NO bioactivity following 1 cup blueberries/d. With effect sizes predictive of 12–15% reductions in CVD risk, blueberries should be included in dietary strategies to reduce individual and population CVD risk. This study was registered at clinicaltrials.gov as NCT02035592.

## Introduction

Metabolic syndrome (MetS) affects approximately one-third of Westernized populations and has been widely reported to increase the risk of type 2 diabetes, cardiovascular (CV) disease, and CV-related mortality. Although insulin resistance is considered central to the development of MetS, compromised vascular function and dysregulated lipids are integral in the etiology, and worsening, of the condition. The clinical management of MetS is initially through lifestyle modifications, with statins and antihypertensive medications added to ongoing lifestyle guidance as MetS severity increases (1). Consequently, identifying effective dietary approaches has clinical relevance throughout MetS progression—as a preventive strategy in nonmedicated individuals, and as an adjunct to those receiving standard pharmacologic therapies.

Blueberries and their main bioactive constituents (especially the flavonoid subclass, anthocyanins) have been identified as candidates to improve CV-related endpoints and the components of MetS. Specifically, prospective studies have shown that higher anthocyanin intakes are associated with lower all-cause mortality (2) and reduced risk of type 2 diabetes (3) and myocardial infarction (4, 5); with benefits also observed for biomarkers including reduced insulin resistance (6) and hypertension (7) and lower weight gain (8). Notably, blueberry intake (ranging from >1 to 3 portions/wk) has been independently associated with many of these benefits (2–4, 8). Major limitations, however, remain with the available randomized controlled trial (RCT) evidence, with blueberry studies being either single-dose/acute interventions (9–12) or short-duration trials (≤8 wk (13–19)), or having provided intakes [e.g., 300 g fresh weight (12), or 45–50 g freeze-dried blueberries/d (16, 17)] that are unlikely to be sustainable in the longer term. Although single-portion studies (10, 11) and continuous feeding over 6–8 wk (13, 16–18) has shown encouraging improvements in vascular function, not all studies report positive vascular outcomes (12, 20), and the effect of blueberry intake on insulin resistance remains equivocal (16, 17).

We investigated the effects of 6-mo blueberry intake (at 2 dietarily achievable levels) on biomarkers of insulin resistance, vascular function, lipid status, and anthocyanin metabolism in adults with MetS.

## Methods

### Study design and participant population

A double-blind, placebo-controlled, parallel study was conducted that enrolled overweight and obese (BMI ≥25 kg/m^2^) adults, aged 50–75 y, with MetS [≥3 MetS components, i.e., impaired fasting glucose, hypertension, central adiposity, hypertriglyceridemia, and low levels of HDL cholesterol (21)], to take part in a 6-mo dietary intervention. Ineligibility included ≤2 MetS components and criteria relating to disease status, lifestyle choices, medication, and supplement use (described in the **Supplemental Methods**), including recent, past, or present smoking history; diabetes, vascular disease, cancer, or digestive, hepatic, or renal disorders; and prescribed hypoglycemic, vasodilator, or hormone-replacement medications. Those with untreated hypertension were excluded at screening. Antihypertensive or statin therapies (or a combination) were permissible following habituation (i.e., antihypertensive medication, ≥6 mo; statins, ≥3 mo).

**FIGURE 1:**
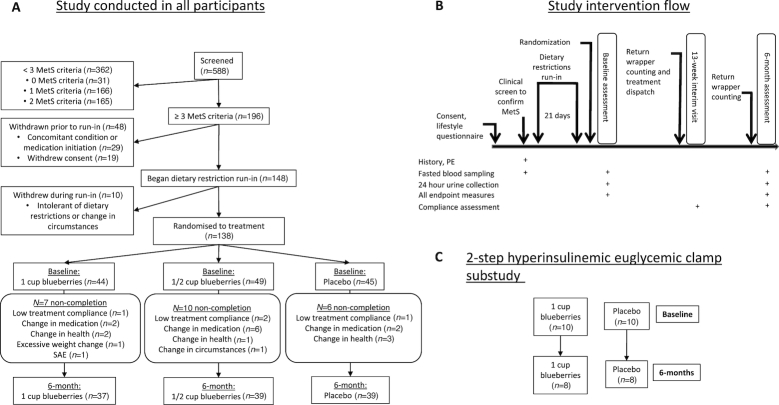
Flow chart of study participants and intervention conduct. (A) Recruitment and retention in the study. (B) An overview of the study involvement for each participant. (C) The flow of study participants in the hyperinsulinemic euglycemic clamp substudy. 1 cup = 150 g blueberries. MetS, metabolic syndrome; SAE, serious adverse event; PE, physical examination.

In total, 138 eligible participants were randomly assigned to 1 of 3 treatment groups, consisting of 2 dietarily achievable blueberry intakes (equivalent to 1 and 1/2 US cup of fresh blueberries per day: 150 and 75 g, respectively) and placebo. AR2007 adaptive random-sequence allocation software (22) was utilized, which incorporated 4 balancing strata that were considered likely a priori to affect insulin resistance and vascular function: sex, number of MetS criteria, age, and statin/blood pressure (BP) medication use. The randomization procedure is further described in Supplemental Methods and allocation to treatment shown in **Figure 1**A. Following a 21-d run-in period of dietary restrictions, insulin resistance and cardiometabolic endpoints were assessed at baseline and 6-mo after intervention (study intervention flow shown in Figure 1B). A substudy of peripheral and hepatic insulin sensitivity was conducted on 20 consenting participants from the main study (Figure 1C), *n* = 10 from each of the 1-cup blueberry and placebo groups.

The primary outcome was change (∆ 0 to 6 mo) in insulin resistance (HOMA-IR). Change (∆ 0 to 6 mo) in vascular function [flow-mediated dilatation (FMD), augmentation index (AIx), carotid-to-femoral pulse wave velocity (cfPWV), and BP], biomarkers of cardiometabolic health [lipid status, nitric oxide (NO) intermediates, glycated hemoglobin (HbA1c), and glucose], and blueberry metabolites were secondary outcomes. The study was approved by the National Research Ethics Committee (East of England) and was conducted between January 2014 and November 2016 at 2 UK Clinical Research Facilities (CRFs): University of East Anglia CRF, and the NIHR/Wellcome Trust CRF (Cambridge University). The study was registered at www.clinicaltrials.gov as NCT02035592, followed the principles of the Declaration of Helsinki of 1975, as revised in 1983, and participants gave written consent before enrollment.

### Intervention products and compliance

In this study, participants were required to consume the intervention product daily for 6 mo. The 3 types of intervention foods (equivalent to 1 and 1/2 US cup blueberries, and placebo) were isocaloric and carbohydrate-matched (glucose 31%, fructose 30%, sucrose 0%), and provided in milled, freeze-dried powder form as follows: 26 g freeze-dried blueberries (1 cup), a hybrid treatment combining 13 g freeze-dried blueberries and 13 g placebo material (1/2 cup), and 26 g placebo (produced by the US National Food Lab). The placebo material's main ingredients were dextrose, maltodextrin, and fructose, which were produced as a purple powder, with blueberry aromatics generated from natural (nonanthocyanin) and artificial color and flavorings. Prior to the study initiation, treatments were tested to be of similar appearance (i.e., color and texture) and matched for taste. Treatment identity was masked by providing all treatments in opaque, 26-g single-serve sachets. Blueberries were from the same harvest (produced by the US Highbush Blueberry Council) and the 1-cup, 1/2-cup, and placebo treatments contained 364, 182, and 0 mg anthocyanin, and 879, 439, and 0 mg phenolics, respectively (levels independently verified in-house). These anthocyanin doses have previously been shown to acutely improve endothelial function (10).

Participants were instructed to consume 1 sachet per d, with 8 standardized recipe ideas provided; these encompassed creating a “blueberry” drink or smoothie, adding the powder to cereals or to yoghurt/desserts, incorporation with banana toast, or adding to salads (top dressing, or within a vinaigrette). The intake method was not prescriptive, to allow participants to incorporate their daily intake into a variety of foods over this extended, 6-mo intervention. Compliance with intervention product intake was calculated from returned wrappers and unused sachets, and the effectiveness of participant blinding was assessed by questionnaire at 6 mo.

### Dietary and lifestyle restrictions

For 21 d prior to, and throughout, the 6-mo study, dietary restrictions were implemented to limit anthocyanin intake (including blueberry abstinence and limits of 1 portion/wk for anthocyanin-rich foods) and other foods known to modify vascular function (see **Supplemental Table 1** and Supplemental Methods for details). Before each assessment visit, strenuous exercise was avoided for 48 h and anthocyanin-rich or nitrate-/nitrite-rich foods, caffeine, and alcohol were avoided for 24 h (see **Supplemental Table 2**). Additionally, low-nitrite/-nitrate bottled water (consumed for 24 h) and a standardized evening meal (anthocyanin-free and low in flavonoids; consumed prior to overnight fasting) was provided. A 131-item validated food-frequency questionnaire (FFQ) (23) was repeated throughout the study (baseline, interim, 6 mo) to monitor adherence to dietary guidance.

### Assessment of insulin resistance and sensitivity, vascular function, and anthropometry

Insulin resistance and insulin sensitivity were calculated from fasting insulin and glucose concentrations through the use of the HOMA-IR calculation and the quantitative insulin sensitivity index (QUICKI), according to standard equations (24, 25). At a separate visit, the 2-step hyperinsulinemic euglycemic clamp substudy was performed at the Cambridge University Hospital CRF as previously described (26); see Supplemental Methods for details of the assessment protocol.

Triplicate BP measurements were taken (separated by 3 min) with an automated sphygmomanometer (Omron 705IT; Omron Healthcare Co.), following 15 min of supine rest in a quiet, temperature-monitored clinical room (21–24°C). Subsequently, 3-lead electrocardiogram gated brachial artery FMD was assessed by ultrasound (Philips iE33; 11–13 MHz linear transducer; Philips) following a standardized procedure: 1 min baseline, 5 min reactive hyperemia (via 220 mmHg sphygmomanometric cuff inflation), 5 min postocclusion (following cuff deflation). Image acquisition and automated edge-detection analysis were performed with commercial software (Vascular Imager and Brachial Analyzer version 5, respectively; Medical Imaging Applications LLC) and percentage FMD (%FMD) was calculated as (diameter_max_ − diameter_baseline_)/diameter_baseline_ × 100. A detailed description of the assessment protocol can be found in the Supplemental Methods.

Aortic distensibility was assessed via cfPWV and systemic arterial stiffness (standardized to a heart rate of 75) was assessed via the AIx (a measure of the wave reflection to arterial pressure waveforms) (both Vicorder, Smart Medical) as previously described (27); ≤6 assessments were made with a target of ≤10% coefficient of variation. Duplicate anthropometric measures (i.e., body weight, height, and waist and hip circumference) were taken. An identical sequence of assessments were performed at baseline and 6 mo.

### Laboratory measurements

After an overnight fast (≥10 h), venous blood was collected and centrifuged, and plasma and serum aliquots were then stored at –80°C. Cardiometabolic biomarkers and anthocyanin/phenolic metabolite concentrations were quantified.

Fasting serum insulin was measured by ELISA (Mercodia) according to the manufacturer's instructions. Fasting glucose, as well as the lipoproteins, i.e., total cholesterol, HDL cholesterol and triglycerides (TGs), were assessed with the use of a clinical chemistry autoanalyzer (ARCHITECT c; Abbott Laboratories) and concentrations of HbA1c were assessed by boronate infinity chromatography (Menarini Hb 9210); both at the Norfolk & Norwich University Hospital. The Friedewald equation (28) was used to calculate LDL cholesterol. To explore the mechanisms underlying the altered lipid and lipoprotein profiles, HDL particle number (HDL-P, *n*), LDL particle number (LDL-P, *n*), apolipoprotein A-I (apoA-I), and apolipoprotein B (apoB) were measured by nuclear magnetic resonance (NMR) spectroscopy (Nightingale Health). Similarly, to evaluate the mechanisms underlying changes in vascular function, plasma nitrite (NO_2_^−^), nitrate (NO_3_^−^), and total nitroso species (RXNO) were measured by ion chromatography and gas-phase chemiluminescence assay (29). Total free thiols were assessed spectrophotometrically following reaction with Ellman's reagent (30). Cyclic guanosine monophosphate (cGMP) was measured by enzyme immunoassay (KGE003; RnD Systems). The intra-assay coefficients of variation were 2.2% (HbA1c), 1.51% (glucose), 0.86% (total cholesterol), 2.91% (HDL cholesterol), 1.23% (TGs), 11.8% (insulin), 1.6% (NO_2_^−^), 16.2% (NO_3_^−^), 15% (RXNO), 10% (total free thiols), and 9.9% (cGMP). The analytical success rate for NMR assessments were 99.6% for apoA-I and apoB and 100% for HDL-P and LDL-P.

In the clamp substudy, fasting plasma insulin (7.65% coefficient of variation) was measured by chemiluminescence immunoassay (Liaison XL model; Diasorin) and plasma nonesterified fatty acid (7.3% coefficient of variation) was assessed by enzymatic colorimetric assay (Roche) (both at Cambridge University Hospitals, Cambridge, UK).

### Anthocyanin-derived phenolic metabolite analysis

Quantification of anthocyanin-derived phenolic acids in serum and 24-h urine samples was performed on an 1200 Agilent HPLC coupled to a SCIEX 3200 Q-trap electrospray ionization tandem mass spectrometer (SCIEX), according to our previously published method (31) with further optimization to include previously reported phenolic metabolites. In total, 72 metabolites were quantified from 3 unique scheduled multiple reaction monitoring methods. A full description can be found in the Supplemental Methods and in **Supplemental Table 3**.

### Statistical analysis

The study sample size was based on a previously reported insulin resistance response following a 6-mo dietary intervention in obese participants (32). A sample size of 117 participants was anticipated to detect a 25% reduction in HOMA-IR (SD 1.1, 90% power, α 0.05); this was increased to 144 participants (*n* = 48/group, allowing recruitment of 24 males/females per group), which accounted for an anticipated ∼20% dropout rate.

Between-group differences in participant characteristics at baseline were assessed by ANOVA or chi-square test. Outliers (±≥3.5 SD from mean) were identified and removed, with a maximum of *n* = 4 outliers identified. Linear mixed-effect models were used to assess the effectiveness of the intervention, including “participant” as a random effect, time, and treatment group, with the time × treatment group interaction taken as the principal analysis of effect [as analyzed in other multidose studies (33)]. Where significant main effects were observed, linear combinations of coefficients between groups were explored. The explanatory variables sex, age, change in BMI, and baseline medication use (statin and antihypertensive medications, where relevant) were included. Nonnormally distributed data (assessed by the skewness–kurtosis test) were analyzed through the use of a generalized linear model with a link log function. For lipid data, a subgroup analysis was conducted in those not prescribed statins.

Data were analyzed with Stata version 14 (Stata Corp.) and *P* and *q* values <0.05 were considered statistically significant. One participant reporting ≤75% treatment compliance and 1 participant with significant bodyweight increase (+9.4 kg, 2.9 kg/m^2^ in 6 mo) were excluded. Completeness of data analysis is further described in the Supplemental Methods.

## Results

In total, 115 participants completed the study (*n* = 37, *n* = 39, *n* = 39; 1 cup (150 g) blueberries, 1/2 cup (75 g) blueberries, and placebo, respectively; see Figure 1A); their baseline characteristics are shown in **Table 1**. Participants were predominantly male (68%), age range 50–75 y, BMI 31.2 kg/m² (range: 25.8–39.6). The predominant number of MetS criteria was 3 (53%) and 4 (40%); MetS cut-off criteria for waist circumference, BP, TG, HDL cholesterol, and glucose were 93%, 80%, 75%, 51%, and 27%, respectively. Statin and antihypertensive medications were prescribed to 38% and 24%, respectively. Intervention adherence was 94.1% compliance across all groups (wrapper return calculations), and 82% of respondents reported an inability to judge treatment allocation. Dietary intake (FFQ) and physical activity did not differ between baseline and 6 mo.

**FIGURE 2:**
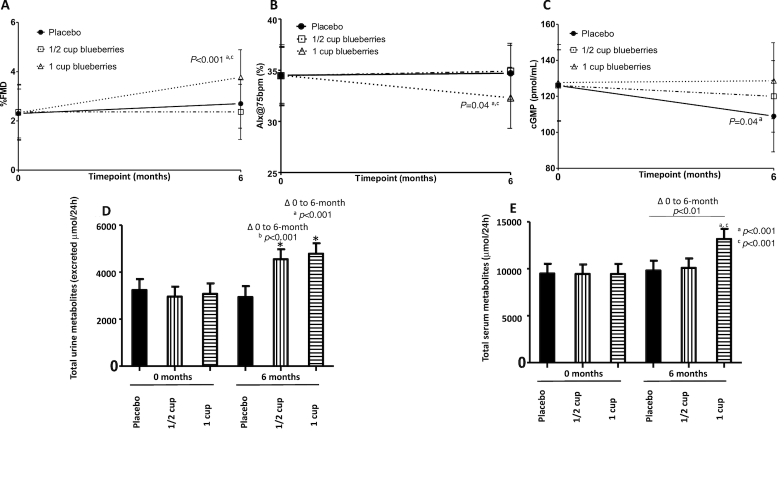
Change in biomarkers of vascular function and total anthocyanin-derived metabolite levels in 24-h urine and serum, from baseline to 6 mo, by intervention group. Change between 0 and 6 mo is reported, by intervention group, for (A) endothelial function (%FMD); (B) arterial stiffness (AIx, standardized at 75 bpm); (C) circulatory levels of cGMP; (D) total urinary anthocyanin-derived metabolites; and (E) total serum anthocyanin-derived metabolites. Values are mean (95% CI). For panels A–C, values are adjusted for baseline values, age, change in BMI, sex, baseline statin intensity, and medication for hypertension. For panels D–E, values are adjusted for sex, sample run plate, baseline BMI, change in fruit and vegetable intake, and use of medications associated with phenolic metabolism. *P* values are shown for the time × treatment interaction calculated from a linear mixed-effect model. Letters indicate significant post-hoc differences between groups; a = 1 cup compared with placebo, b = 1/2 cup compared with placebo, and c = 1 cup compared with 1/2 cup. Participant numbers: A, 1 cup (*n* = 29), 1/2 cup (*n* = 33), placebo (*n* = 34); B, 1 cup (*n* = 35), 1/2 cup (*n* = 39), placebo (*n* = 38); C, 1 cup (*n* = 36), 1/2 cup (*n* = 38), placebo (*n* = 39); D, 1 cup (*n* = 28), 1/2 cup (*n* = 27), placebo (*n* = 31); E, 1 cup (*n* = 32), 1/2 cup (*n* = 34), placebo (*n* = 37). 1 cup = 150 g blueberries. AIx@75 bpm, augmentation index standardized to 75 heart beats per minute; cGMP, cyclic guanosine monophosphate; FMD, flow-mediated dilatation.

**TABLE 1:** Participant characteristics at baseline for the 115 adults with MetS who completed the 6-mo, multidose, freeze-dried blueberry intervention trial^1^

	All (*n* = 115)	Placebo (*n* = 39)	1/2 cup blueberries (*n* = 39)	1 cup blueberries (*n* = 37)
Age, y	62.8 ± 7.1	62.9 ± 8.1	62.6 ± 7.2	63.0 ± 5.9
Gender (M), *n* (%)	78 (67.8)	26 (66.7)	28 (71.8)	24 (64.9)
BMI, kg/m^2^	31.2 ± 3.0	31.1 ± 3.0	31.2 ± 2.6	31.3 ± 3.4
HbA1c, % (mmol/mol)	5.7 ± 0.31 (39.0 ± 3.4)	5.7 ± 0.31 (38.7 ± 3.4)	5.7 ± 0.30 (39.2 ± 3.3)	5.7 ± 0.33 (39.2 ± 3.6)
Hypertension medication, *n* (%)	28 (24.3)	10 (25.6)	9 (23.1)	9 (24.3)
Hyperlipidemia medication, *n* (%)	44 (38.3)	16 (41.0)	15 (38.5)	13 (35.1)
No. of MetS criteria confirmed at screening, *n* (%)				
3 criteria	61 (53.0)	22 (56.4)	21 (53.8)	18 (48.6)
4 criteria	46 (40.0)	16 (41.0)	16 (41.0)	14 (37.8)
5 criteria	8 (7.0)	1 (2.6)	2 (5.1)	5 (13.5)
FFQ dietary intake at baseline				
Energy, kcal/d	1971 ± 555	1875 ± 517	2002 ± 595	2038 ± 551
Fat, % energy	35.1 ± 4.5	34.5 ± 5.1	35.6 ± 4.5	35.1 ± 3.9
Carbohydrate, % energy	43.0 ± 6.4	44.5 ± 5.9	41.9 ± 7.5	42.6 ± 5.4
Protein, % energy	18.5 ± 3.4	17.9 ± 3.1	18.6 ± 3.7	19.1 ± 3.5
Anthocyanins, mg/d	18.6 ± 13.6	18.6 ± 12.6	16.6 ± 14.8	20.8 ± 13.3

^1^Values are mean ± SD or *n* (%). Dietary data excluded for *n* = 6 considered invalid (placebo, *n* = 3; 1/2 cup, *n* = 1; 1 cup, *n* = 2). HbA1c data missing for *n* = 1 placebo. 1 cup = 150 g blueberries. FFQ, food-frequency questionnaire; HbA1c, glycated hemoglobin; MetS, metabolic syndrome.

No favorable effects of the intervention were shown for the primary endpoint HOMA-IR or indices of glucose control [QUICKI, HbA1c (**Table 2**)] and peripheral, hepatic, and adipose tissue insulin sensitivity was unchanged (confirmed by clamp assessment in a subgroup) (**Supplemental Table 4**). However, after 6 mo of 1 cup blueberries/d %FMD significantly increased (1 cup: 1.45%; 1/2 cup: 0.00%; placebo: 0.39%; *P* = 0.003), and AIx significantly reduced, compared with other treatments (1 cup: –2.24%; 1/2 cup: 0.45%; placebo: 0.24%; *P* = 0.04) (**Figure 2**A, B). Similarly, mean plasma cGMP concentrations were increased following 1-cup intake (1 cup: 0.99 pmol/mL; 1/2 cup: –6.15 pmol/mL; placebo: –16.75 pmol/mL) and the time × treatment interaction between groups was significant (*P* = 0.04) (Figure 2C). The intervention had no effect on BP (Table 2) or other biomarkers of vascular function and systemic redox status (total free thiols) (**Supplemental Table 5**).

**TABLE 2:** Change in insulin resistance and glucose homeostasis, blood pressure, and lipid levels from baseline to 6 mo by intervention group^1^

	Placebo (*n* = 39)			1/2 cup blueberries (*n* = 39)			1 cup blueberries (*n* = 37)			
	Before	After	∆ 0 to 6 mo	Before	After	∆ 0 to 6 mo	Before	After	∆ 0 to 6 mo	*P*
HOMA-IR^2^	2.0 (1.9, 2.2)	2.1 (1.9, 2.2)	0.07 (–0.12, 0.26)	1.9 (1.8, 2.1)	2.3 (2.1, 2.4)	0.32 (0.15, 0.49)	2.0 (1.9, 2.2)	2.1 (1.9, 2.2)	0.05 (–0.13, 0.24)^c^	0.07
QUICKI^2^	0.35 (0.34, 0.35)	0.34 (0.34, 0.35)	0.00 (–0.01, 0.00)	0.35 (0.34, 0.35)	0.34 (0.34, 0.34)	–0.01 (–0.01, 0.00)	0.35 (0.34, 0.35)	0.34 (0.34, 0.35)	0.00 (–0.01, 0.00)	0.55
Glucose,^2^ mmol/L	5.2 (5.2, 5.3)	5.2 (5.1, 5.3)	–0.04 (–0.13, 0.06)	5.2 (5.2, 5.3)	5.3 (5.2, 5.4)	0.06 (–0.03, 0.16)	5.2 (5.2, 5.3)	5.2 (5.1, 5.3)	–0.03 (–0.13, 0.07)	0.24
Insulin,^2^ mU/L	9.0 (8.4, 9.6)	9.8 (9.2, 10.4)	0.78 (–0.12, 1.7)	9.0 (8.3, 9.6)	10.6 (9.9, 11.2)	1.6 (0.73, 2.5)	9.1 (8.4, 9.7)	9.7 (9.1, 10.4)	0.66 (–0.28, 1.6)	0.28
HbA1c,^2^ % (mmol/mol)	5.7 (5.7, 5.8); [39.1 (38.6, 39.6)]	5.7 (5.7, 5.8); [39.3 (38.7, 39.8)]	0.01 (–0.06, 0.09); [0.16 (–0.61, 0.93)]	5.7 (5.7, 5.8); [39.1 (38.6, 39.7)]	5.8 (5.7, 5.8); [39.9 (39.3, 40.4)]	0.07 (0.00, 0.14); [0.76 (–0.01, 1.5)]	5.7 (5.7, 5.8); [39.1 (38.6, 39.7)]	5.8 (5.7, 5.8); [39.5 (38.9, 40.0)]	0.03 (–0.04, 0.10); [0.36 (–0.42, 1.1)]	0.55
Systolic BP,^2^ mmHg	136 (134, 138)	133 (131, 135)	–2.58 (–5.51, 0.36)	136 (134, 138)	135 (133, 137)	–0.63 (–3.56, 2.3)	136 (134, 138)	134 (132, 136)	–1.50 (–4.55, 1.6)	0.65
Diastolic BP,^2^ mmHg	81.2 (79.9, 82.5)	82.3 (81.0, 83.6)	1.1 (–0.69, 2.9)	80.9 (79.6, 82.2)	82.2 (80.9, 83.5)	1.3 (–0.53, 3.1)	81.6 (80.3, 83.0)	80.9 (79.5, 82.2)	–0.75 (–2.64, 1.1)	0.24
Cholesterol, mmol/L	5.4 (5.2, 5.5)	5.4 (5.3, 5.5)	0.02 (–0.16, 0.21)	5.4 (5.3, 5.6)	5.5 (5.3, 5.6)	0.02 (–0.17, 0.20)	5.4 (5.3, 5.5)	5.6 (5.4, 5.7)	0.19 (0.00, 0.38)	0.35
LDL cholesterol, mmol/L	3.4 (3.3, 3.5)	3.4 (3.3, 3.5)	0.04 (–0.11, 0.20)	3.4 (3.3, 3.6)	3.4 (3.2, 3.5)	–0.09 (–0.24, 0.07)	3.4 (3.3, 3.5)	3.5 (3.4, 3.6)	0.09 (–0.06, 0.25)	0.24
HDL cholesterol, mmol/L	1.2 (1.1, 1.2)	1.1 (1.1, 1.2)	–0.02 (–0.06, 0.02)	1.2 (1.1, 1.2)	1.2 (1.1, 1.2)	0.00 (–0.04, 0.04)	1.2 (1.1, 1.2)	1.2 (1.2, 1.2)	0.04 (0.00, 0.08)^a^	0.08
Total cholesterol:HDL cholesterol	4.7 (4.6, 4.8)	4.7 (4.6, 4.8)	0.06 (–0.11, 0.22)	4.7 (4.6, 4.8)	4.7 (4.6, 4.9)	0.06 (–0.11, 0.22)	4.7 (4.6, 4.8)	4.7 (4.6, 4.8)	–0.01 (–0.18, 0.16)	0.83
TGs, mmol/L	1.7 (1.6, 1.8)	1.7 (1.6, 1.8)	–0.03 (–0.17, 0.12)	1.7 (1.6, 1.8)	2.0 (1.9, 2.1)	0.24 (0.10, 0.38)^b^	1.8 (1.6, 1.9)	1.9 (3.4, 3.6)	0.10 (–0.04, 0.25)	0.04

1 Values are mean (95% CI) adjusted for baseline values, age, change in BMI, sex, baseline statin intensity. Cup indicates the equivalent number of US cups of fresh blueberries. *P* values are for the time × treatment interaction calculated with the use of a linear mixed-effect model. Letters indicate significant differences between groups: a = 1 cup compared with placebo, b = 1/2 cup versus placebo, and c = 1 cup compared with 1/2 cup. 1 cup = 150 g blueberries. BP, blood pressure; HbA1c, glycated hemoglobin; QUICKI, quantitative insulin sensitivity index; TG, triglyceride.

2 A maximum of *n* = 4 outliers were identified. Exclusion from systolic and diastolic BP assessment due to change in BP medication during study, *n* = 1.

**FIGURE 3:**
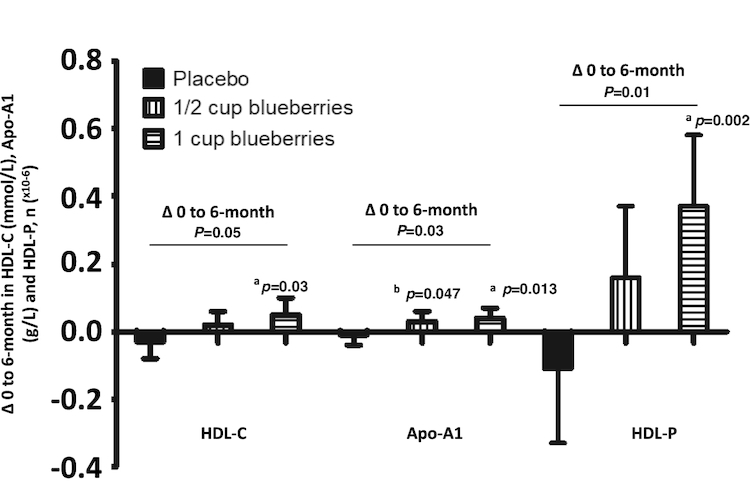
Changes in HDL cholesterol, HDL particle number and apoA-I levels from baseline to 6 mo by statin nonusers (*n* = 71), by intervention group. Change between 0 and 6 mo, reported by intervention group, for levels of HDL cholesterol (mmol/L) and apoA-I (g/L) and the number of HDL particles (×10^–6^) in volunteers with MetS, and not medicated with statins, after 6-mo daily intake of 1/2 or 1 cup (75 g or 150 g) blueberries or matched placebo. Values are mean (95% CI), adjusted for baseline values, age, change in weight, sex. *P* values are shown for the time × treatment interaction calculated from a linear mixed-effect model. Letters indicate significant differences between groups: a = 1 cup compared with placebo, b = 1/2 cup compared with placebo and c = 1 cup compared with 1/2 cup. Participant numbers: 1 cup (*n* = 24), 1/2 cup (*n* = 23), placebo (*n* = 24); *n* = 1 outlier excluded (1 cup group) for apoA-I analysis. apoA-I, apolipoprotein A-I; MetS, metabolic syndrome.

Relative to placebo, 1 cup increased HDL cholesterol levels (1 cup: + 0.04 mmol/L; placebo: –0.02 mmol/L; *P* = 0.03), with a trend towards a dose-related increase (*P* = 0.08) (Table 2). When statin users were excluded (*n* = 44; 38% of population), a significant 0.08-mmol/L net difference in HDL cholesterol concentrations was observed between the 1-cup group (+0.05 mmol/L) and the placebo group (–0.03 mmol/L) (*P* < 0.03; **Figure 3**). NMR spectroscopy analysis in statin nonusers showed that both apoA-I and HDL-P, *n* were significantly increased (∆ 0 to 6 mo) in the 1-cup group (Figure 3) compared with placebo (*P* = 0.002 and *P* = 0.013, respectively).

The intervention had no effect on total cholesterol and LDL cholesterol levels or the total cholesterol:HDL cholesterol ratio. However, TG levels differed (*P* = 0.04; Table 2), with a significant increase in the 1/2-cup blueberry group compared with placebo (*P* = 0.01, Table 2); this remained when analysis was restricted to statin nonusers (*P* = 0.04; **Supplemental Table 6**). In further analysis in statin nonusers, NMR data showed that neither LDL-P, *n* nor apoB were significantly altered following the 1/2-cup blueberry intake (Supplemental Table 6).

In serum and 24-h urine, total concentrations of anthocyanin-derived phenolic acid metabolites significantly increased (∆ 0 to 6 mo) following blueberry intake (*P* < 0.01 and *P* < 0.001, respectively; in a dose-dependent manner, compared with placebo) (**Table 3** and Figure 2D, E). Individual metabolites that changed were almost exclusively by-products of microbial catabolism and human phase II metabolism (Table 3).

**TABLE 3:** Significant changes in anthocyanin-related metabolites and phase II conjugates in serum and urine, from baseline to 6 mo: results presented by intervention group and concentrations presented for summed serum and urine totals, and individual metabolites and phase II conjugates^1^

	∆ 0 to 6 mo: placebo	∆ 0 to 6 mo: 1/2 cup	∆ 0 to 6 mo: 1 cup	*P*
Serum metabolites,^2^ nmol/L	*n* = 38	*n* = 34	*n* = 32	
Total serum metabolites	325 (–1141, 1791)^a^	645 (–761, 2050)	3722 (2211, 5234)^c^	<0.01
Hippuric acid	–244 (–546, 57.5)^a^	1193 (822, 1563)^b^	2468 (1892, 3045)^c^	<0.001
3/4-Methoxybenzoic acid-3/4-sulfates^3^	–0.16 (–3.3, 2.9)^a^	1.9 (–1.0, 4.9)	10.4 (7.0, 13.9)^c^	<0.01
4-Hydroxy-3-methoxyphenylpropionic acid	1.4 (–1.7, 4.4)	–2.6 (–4.8, –0.32)	5.4 (2.7, 8.1)^c^	<0.01
2,6-Dihydroxybenzoic acid	–6.0 (–14.1, 2.0)	9.4 (0.86, 18.0)	–12.2 (–19.0, –5.5)^c^	<0.01
3,4-Dihydroxycinnamic acid	–3.4 (–11.0, 4.2)	7.8 (1.9, 13.6)	–7.5 (–12.4, –2.7)^c^	<0.01
2-Hydroxybenzoic acid	–56.1 (–83.6, –28.6)^a^	–22.8 (–49.5, 3.8)	15.5 (–13.8, 44.8)	0.02
3,5-Dihydroxybenzyl alcohol	2.8 (–10.6, 16.2)	–22.1 (–31.3, –13.0)^b^	–8.4 (–17.6, 0.80)	0.03
4-Hydroxy-3,5-dimethoxyphenylacetic acid	0.94 (–4.7, 6.6)	5.2 (–0.16, 10.5)	–6.8 (–11.5, –2.0)^c^	0.03
Urine metabolites,^2^ µmol in 24 h	*n* = 31	*n* = 27	*n* = 29	
Total urine metabolites	–300 (–928, 327)^a^	1593 (1007, 2179)^b^	1707 (1091, 2324)	<0.001
Chlorogenic acid	–0.07 (–0.10, –0.04)^a^	0.13 (0.09, 0.17)^b^	0.23 (0.16, 0.30)	<0.001
4-Hydroxy-3,5-dimethoxybenzoic acid (syringic acid)	–0.03 (–0.12, 0.07)^a^	0.41 (0.31, 0.51)^b^	0.74 (0.61, 0.88)^c^	<0.001
Benzoylglutamic acid	–0.10 (–0.26, 0.06)^a^	0.86 (0.64, 1.1)^b^	0.86 (0.63, 1.1)	<0.001
4-Hydroxy-3-methoxybenzoic acid (vanillic acid)	–1.6 (–2.2, –1.1)^a^	–0.85 (–1.4, –0.31)	1.3 (0.57, 2.0)^c^	<0.001
Hippuric acid	–262 (–693, 168)^a^	1261 (859, 1662)^b^	1579 (1157, 2002)	<0.001
4-Methoxybenzoic acid-3-glucuronide (isovanillic acid-3-glucuronide)	–0.17 (–0.27, –0.08)^a^	–0.08 (–0.15, –0.02)	0.32 (0.19, 0.45)^c^	<0.001
2-Hydroxybenzoic acid	–0.10 (–0.21, 0.01)^a^	0.41 (0.30, 0.53)^b^	0.14 (0.03, 0.25)^c^	<0.001
4-Hydroxy-3-methoxycinnamic acid	–0.07 (–0.19, 0.04)^a^	0.15 (0.05, 0.25)^b^	0.49 (0.37, 0.62)^c^	<0.001
3,4-Dihydroxyphenylpropionic acid (dihydrocaffeic acid)	–0.29 (–0.50, –0.09)^a^	0.15 (–0.10, 0.40)^b^	0.60 (0.34, 0.85)	<0.001
*Trans*-3-hydroxycinnamic acid (*m*-coumaric acid)	–0.04 (–0.08, 0.01)^a^	0.05 (0.01, 0.10)^b^	0.13 (0.08, 0.19)	<0.001
3-Hydroxyhippuric acid	–11.6 (–29.2, 6.1)^a^	29.3 (12.4, 46.2)^b^	55.6 (36.0, 75.3)	<0.001
3-Hydroxy-4-methoxycinnamic acid	–0.05 (–0.08, –0.02)^a^	0.04 (0.01, 0.06)^b^	0.02 (–0.01, 0.04)	<0.001
3/4-Hydroxybenzoic acid-3/4-sulfates^3^	0.09 (–0.40, 0.59)^a^	1.6 (1.0, 2.2)^b^	2.0 (1.4, 2.6)	<0.001
2,5-Dihydroxybenzoic acid	–0.58 (–1.2, 0.04)^a^	1.2 (0.58, 1.8)^b^	0.94 (0.33, 1.6)	<0.001
3,4-Dihydroxyphenylacetic acid	–0.11 (–0.81, 0.60)^a^	0.72 (0.07, 1.4)	1.9 (1.2, 2.6)	<0.01
3-Hydroxybenzoic acid	–0.22 (–0.27, –0.16)	–0.02 (–0.13, 0.10)^b^	–0.17 (–0.22, –0.13)	<0.01
3,5-Dimethoxybenzoic acid methyl ester	–0.02 (–0.03, –0.01)^a^	0.01 (–0.01, 0.04)	0.02 (0.00, 0.03)	<0.01
4-Hydroxyhippuric acid	–6.1 (–15.7, 3.5)^a^	–9.9 (–18.2, –1.7)	13.5 (3.2, 23.7)^c^	<0.01
3-Hydroxyphenylpropionic acid	0.03 (–0.16, 0.22)^a^	0.34 (0.14, 0.54)	0.97 (0.58, 1.4)^c^	<0.01
4-Hydroxy-3,5-dimethoxycinnamic acid	–0.02 (–0.14, 0.11)	–0.03 (–0.10, 0.04)	0.20 (0.10, 0.30)^c^	<0.01
4-Hydroxy-3-methoxyphenylacetic acid	–3.3 (–5.7, –0.97)^a^	2.1 (–0.46, 4.6)^b^	1.4 (–1.1, 4.0)	0.01
3,4-Dihydroxycinnamic acid	0.04 (–0.06, 0.14)	0.10 (0.02, 0.17)	–0.09 (–0.18, 0.00)^c^	0.03
3,4,5-Trimethoxybenzaldehyde	0.00 (–0.01, 0.01)	–0.02 (–0.03, –0.01)^b^	0.00 (–0.01, 0.02)	0.03
3/4-Methoxybenzoic acid-3/4-sulfates^3^	–0.12 (–3.0, 2.7)	6.0 (3.6, 8.4)^b^	1.8 (–0.42, 4.1)	0.03
4-Hydroxy-3-methoxyphenylpropionic acid	–0.54 (–1.1, 0.03)^a^	–0.46 (–0.89, –0.03)	0.60 (–0.05, 1.2)	0.03

^1^Values are mean (95% CI) adjusted for sex, sample run plate, baseline BMI, change in fruit and vegetable intake, and use of medications associated with phenolic metabolism. Cup indicates the equivalent number of US cups of fresh blueberries. Outliers < or >3.5 SD were excluded. *P* is the false discovery rate–adjusted value for the time × treatment interaction calculated with the use of a linear mixed-effect model; only significant metabolites are shown; letters indicate significant differences between groups: a = 1 cup compared with placebo, b = 1/2 cup compared with placebo, and c = 1 cup compared with 1/2 cup (based on false discovery rate–adjusted *P* values). 1 cup = 150 g blueberries.

^2^Across urine and serum analysis, a maximum of *n* = 3 outliers were identified in any metabolite assessment.

^3^Where two compounds are reported together, they could not be sufficiently resolved.

## Discussion

In the longest-duration blueberry RCT to date, conducted in 115 adults with MetS, we report evidence for the following: (i) 1 cup (150 g) blueberries/d for 6 mo resulted in sustained and clinically relevant improvements in endothelial function, systemic arterial stiffness, and HDL cholesterol concentrations (especially in statin nonusers); (ii) increased cGMP levels, HDL cholesterol particle density, and apoA-I levels are likely to be underlying improvements in vascular and lipid status; and (iii) insulin resistance and peripheral, hepatic, and adipose tissue insulin sensitivity were unchanged by blueberry intake.

Despite finding no benefit in terms of insulin resistance, our data demonstrate that higher intakes of blueberries (∼1 cup/d) improved markers of vascular function and elements of lipid status. Over 6 mo, 1 cup blueberries/d improved conduit artery endothelial function (assessed by %FMD; effect size of 1.06% compared with placebo) by a magnitude that translates to a 13% reduction in future CV events based on previous meta-analyses (34). Furthermore, we observed reduced systemic arterial stiffness (assessed by AIx) and also identified improvements in HDL cholesterol, which were most pronounced in statin nonusers. Notably, the 3.09-mg/dL (0.08-mmol/L) difference in HDL cholesterol between statin nonusers (when comparing 1 cup/day with placebo) would equate to 6.2–9.3% lower risk of coronary heart disease 11.4–14.5% lower risk of CV disease (men and women, respectively) based on predictive data from prospective studies (35). There has been a long-established synergy between endothelial dysfunction and arterial stiffness (36), and between AIx and hypercholesterolemia (37, 38), and our data identify 1 cup (150 g)/d as an effective dietary approach to reduce CV risk.

The observed increase in cGMP concentrations provides a potential mechanistic insight into the observed cardiometabolic effects, with a dose-dependent effect on cGMP concentrations following blueberry/anthocyanin intake. These data suggest that circulating cGMP levels reflect the activity status of soluble guanylate cyclase in vascular smooth muscle coupling, which is stimulated by endothelial NO. In support of this, previous evidence of increased plasma cGMP following 12 wk of purified anthocyanin extract consumption (39) adds weight to the suggestion that the anthocyanins may be responsible for this effect. Contrary to our expectations, but consistent with blueberry interventions lasting 6–8 wk (14, 18), cGMP increases were associated neither with concomitant elevations in NO metabolite levels (excluding the possibility of increased tonic endothelial NO formation) nor with plasma thiol availability (indicating no gross changes in extracellular redox status). Taken together, this biomarker signature is consistent with an enhanced NO bioavailability, potentially mediated by the anthocyanin-induced increase in plasma superoxide dismutase activity, as reported recently (40). NMR analysis also confirmed significantly increased HDL particle number and apoA-I levels following 1 cup blueberries/d, which likely underpin the clinically relevant increase in HDL cholesterol. Recently, the JUPITER study confirmed that HDL particle number was the only predictor of incident CV disease (41), and the China Kadoorie Biobank prospective study established that a 1-SD increase in very large, large, and medium HDL cholesterol particles significantly reduced myocardial infarction risk (13% and 20% reduction in risk), and a 1-SD increase in apoA-I reduced myocardial infarction risk by 11% (42).

Prior to our long-term study, only acute improvements in FMD were reported following single-dose blueberry intake (10, 11), and only microvascular endothelial function was improved in MetS after short-term (6-wk) blueberry intake (16); the latter was assessed by finger tonometry, which has been shown to be poorly correlated with CVD risk markers/atherosclerosis or FMD (43, 44). Likewise, the benefits of blueberries on arterial stiffness were previously limited to healthy younger adults (13) and postmenopausal women (with pre- and stage 1 hypertension) (18), in relatively short-term studies (6–8 wk).

Although meta-analyses of prospective cohort studies have observed a reduced risk of diabetes with higher habitual anthocyanin (45) or blueberry intake (46), not all of the included studies have identified benefits (47). Similarly, RCTs to date have not consistently supported effects on intermediate markers associated with diabetes development, such as insulin resistance or glucose metabolism. Despite 1 short-term study (6 wk) showing that blueberries (668 mg anthocyanins) improved insulin sensitivity in obese patients with insulin resistance (15), others have reported increased HbA1c and HOMA-IR following 8-wk blueberry intake [742 mg anthocyanins (17)] in hypertensive postmenopausal women, and no effect on insulin sensitivity in patients with MetS (581 mg anthocyanins) (16). We found no benefit of blueberries on any of our multi-endpoint insulin resistance analyses, and although *n* = 115 completed the study, compared with the *n* = 117 in our HOMA-IR power calculation, it is considered unlikely that this would materially affect our study findings. In our gold-standard 2-step, glucose tracer–labeled, hyperinsulinemic euglycemic clamp we further confirmed that peripheral, hepatic, and adipose tissue insulin sensitivity was unaffected and there was no suppression of hepatic glucose production and adipose tissue lipolysis. The lack of effect in our study may relate to the high-risk participants under investigation, with a greater likelihood of a benefit in healthy participants. The prospective data have follow-up over a number of years, and frequent habitual intake over the long term may be necessary to observe a benefit on incident disease.

In keeping with a meta-analysis of 6 previous blueberry interventions, we observed no change in BP (48) and, as it is generally accepted that aortic distensibility (PWV) is strongly associated with peripheral BP (49), it was unsurprising that cfPWV also remained unchanged (Table 2). Unexpectedly, we found no benefit of a smaller daily intake of blueberries (∼1/2 cup/d, providing 182 mg anthocyanins) and, in the case of TG, this level of blueberry intake resulted in a modest increase; a finding contrary to a recent meta-analysis showing benefits of short-term anthocyanin supplementation trials (45 d–24 wk) on TG and other lipid fractions, in patients with dyslipidaemia (50). The lack of effect in the 1/2-cup group warrants further speculation. Although health benefits have been associated with long-term, but modest, habitual blueberry intakes (2, 5), 1/2 cup/d for 6 mo may be insufficient to provide chronic benefits in an obese, at-risk population. Relative to previous short-duration blueberry RCTs (providing ∼335–742 mg anthocyanins/d) (13, 17), our daily 1/2 cup of blueberries provided lower anthocyanin intakes (of 182 mg/d), which resulted in significantly lower concentrations of total serum metabolites than the 1-cup group; we reason, based on animal data showing that anthocyanin intake profoundly affects gut microbial community structure leading to improved biomarkers of cardiometabolic risk (51, 52), that the lower serum metabolite concentrations and metabolite profile variability (identified in urine) may, in part, be responsible for a lack of an intervention effect in our 1/2-cup group.

Following ingestion, anthocyanins undergo extensive metabolism, most of which occurs in the lower intestine. These metabolites therefore serve as growth substrates for the gut microbiome and likely play a key beneficial metabolic role (53). In our study, we observed an increase in the combined total concentration of microbially derived anthocyanin metabolites and their human phase II conjugates, in both serum and 24-h urine, following blueberry intake. These increases were observed in a blueberry-dose–dependent manner and support the high compliance with intervention in our study, further indicated by wrapper returns. Eight metabolites in serum and 25 metabolites in 24-h urine differed significantly between intervention groups, some of which have previously been shown to have cardiometabolic bioactivity in animal and in vitro studies. For example, 4-hydroxy-3,5-dimethoxybenzoic acid (syringic acid) has increased endothelial nitric oxide synthase expression and NO levels in cultured human endothelial cells (54, 55), whereas 3,4-dihydroxyphenylpropionic acid (dihydrocaffeic acid) and 4-hydroxy-3-methoxybenzoic acid (vanillic acid) levels were associated with improved cardiometabolic health in animal models of hypercholesterolemia (56) and hypertension (57). Human data are now required to understand the contribution of individual metabolites, or combinations of metabolites (“metabolic signatures”), to cardiometabolic health.

The long duration of our intervention is a particular strength as our study data have narrowed the gap in understanding between previous acute (10, 11) and short-term RCTs (13, 16–18) and the extensive prospective data which show that higher habitual intakes of blueberries/anthocyanins reduce CVD risk (2, 4, 5, 8). Our selection of participants with MetS addresses an underrepresented at-risk population and our use of an extensive range of gold-standard methodologies (i.e., FMD and clamp), supported by highly specific NO/redox biomarkers and NMR lipid analyses, has robustly assessed the effectiveness of the intervention. A potential limitation is that despite an open recruitment policy, our population was predominantly white and those with confirmed MetS (at screening) were predominantly men, reducing our ability to establish whether these data translate to other ethnicities and if gender disparities exist.

In conclusion, we present what we believe to be the first longer-term evidence of clinically relevant improvements in endothelial function and systemic arterial stiffness, following blueberry intake, in an at-risk population with MetS; most likely due to improvements in NO bioactivity and lipid status. The simple and attainable message to consume 1 cup of blueberries daily should be given to those aiming to improve their CV health.

## Supplementary Material

nqy380_Supplemental_File

## Notes

Supported by the US Highbush Blueberry Council (USHBC) with oversight from the USDA and the Biotechnology and Biological Sciences Research Council (BBSRC, UK). AC and ERB both act as advisors to the USHBC grant committee. The funders of the study had no role in study design, data collection, data analysis, data interpretation, or writing of the report.

Supplemental Methods and Supplemental Tables 1–6 are available from the “Supplementary data” link in the online posting of the article and from the same link in the online table of contents at https://academic.oup.com/ajcn/.

Abbreviations used: AIx, augmentation index; apoA-I, apolipoprotein A-I; apoB, apolipoprotein B; BP, blood pressure; cfPWV, carotid-to-femoral pulse wave velocity; CRF, Clinical Research Facility; CV, cardiovascular; CVD, cardiovascular disease; cGMP, cyclic guanosine monophosphate; FFQ, food-frequency questionnaire; FMD, flow-mediated dilatation; HbA1c, glycated hemoglobin; HDL-P, *n*, high-density lipoprotein particle number; LDL-P, *n*, low-density lipoprotein particle number; MetS, metabolic syndrome; NMR, nuclear magnetic resonance; NO, nitric oxide; QUICKI, quantitative insulin sensitivity index; RCT, randomized controlled trial; RXNO, *S*-nitrosothiols + other nitroso species; TG, triglyceride.
