# Supplementary material for: Blueberries improve biomarkers of cardiometabolic function in participants with metabolic syndrome—results from a 6-month, double-blind, randomized controlled trial
Source: Am J Clin Nutr. 2019 May 28;109(6):1535–45. doi: 10.1093/ajcn/nqy380 (PMC6537945; doi:10.1093/ajcn/nqy380)
Supplement: nqy380_Supplemental_File [file nqy380_supplemental_file.docx]

**ONLINE-ONLY SUPPLEMENTAL MATERIAL**

**SUPPLEMENTAL ABBREVIATIONS:** AIx, Augmentation index; Apo-A1, apolipoprotein A1; Apo-B, apolipoprotein B; BP, Blood pressure; cfPWV, Carotid to femoral pulse wave velocity; CRF, clinical research facility; ECG, electrocardiogram; FDR, false discovery rate; FMD; flow mediated dilatation; GCMS, gas chromatography-mass spectrometry; HDL-P, high density lipoprotein particles; LDL-P, low density lipoprotein particles; LOD, limit of detection; MetS, metabolic syndrome; MCR, metabolic clearance rate; NEFA, non-esterified fatty acids; NMR, nuclear magnetic resonance; Ra, rate of glucose appearance; Rd, rate of glucose disposal; RXNO, S-nitrosothiols + other nitroso species; SPE, solid phase extraction; TG, triglycerides.

**SUPPLEMENTAL METHODS**

*Inclusion and exclusion criteria:*

Ineligibility included BMI<25kg/m^2^, being <50 years or ≥76 years of age, having ≤ 2 MetS components confirmed at the clinical screen, as well as the following; history of recent, past or present smoking status; presence of diabetes, vascular disease, cancer, or digestive, hepatic, renal, haematological, neurological and thyroid disorders; prescribed use of hypoglycaemic, vasodilator, or hormone replacement medications, selective serotonin reuptake inhibitors, anti-gout and anti-psychotics. Similarly, habitual users of analgesia (including, ≥4d/wk. of non-steroidal anti-inflammatories) and antihistamines were ineligible. Those with untreated hypertension were excluded at screening. The ethics committee approved the researchers to recruit those on established anti-hypertensive medication on 29JUL2014 to widen the inclusivity of the research; as standard frontline management is to consider / prescribe anti-hypertensive medications to overweight / obese adults, ≥ 50 yr., presenting with raised BP. To ensure that there were no transient effects of anti-hypertensive and/or statin therapy initiation on the study results, inclusion of those on these medications was permissible only following habituation (i.e. anti-hypertensive medication, ≥6-month; statins, ≥3-month). Whilst supplements containing flavonoids were not permitted, other dietary supplements (such as fish oils) were permissible if sustained throughout the study.

*Randomization to treatment and blinding*

An administrator, with no data collection or analysis responsibility, maintained the adaptive random-sequence allocation dataset (uploading volunteer sex, number of metabolic syndrome criteria at screening, age and statin/BP medication use) and generated the randomisation sequence of participants to intervention. A unique numeric code was assigned to each participant (generated, and only accessible to the administrator), which was linked to the alphabetical study treatments A, B and C. Only ERB and AJ (not involved with the study conduct and assessment) possessed the information to link the alphabetical identifier with treatment identity. Researchers assessing outcomes and analysing biological samples, and clinical staff, were blind to treatment throughout the study and statistical analysis.

*Dietary and lifestyle modifications*

A number of dietary restriction were implemented to limit the intake of food rich in anthocyanins and other foods known to modify vascular function. For 21d prior to, and throughout the 6-month study, blueberry intake was not permissible (including within composite foods and also in products made from blueberry) and a limit of one portion per week was also instigated for other anthocyanin-rich foods. In addition, a limit on the intake of dark chocolate (two, 5 chunk portions /wk.), oily fish (two medium portions /wk.), red wine (one, 125ml portion /wk.), tea and coffee (combined maximum of 4 cups/d) was set and UK alcohol guidelines were advocated (≤14 and 21 units/wk. for women and men respectively). To facilitate these restrictions, a full list of foods to exclude was provided (see Supplemental **Table S1**). During periods when low nitrite/nitrate intakes were required (i.e. within 24h of assessment visits) a further list of prohibited foods were provided to participants (see Supplemental **Table S2**). A checklist was completed at the start of each visit to confirm adherence to the dietary restrictions. Because participants were instructed to maintain their habitual activity levels throughout the study, the international physical activity questionnaire (long last 7d format version (1)) was administered at baseline, interim (week 13) and 6 months.

*2-step hyperinsulinemic euglycemic clamp method and data analysis*

In the 2-step hyperinsulinemic euglycemic clamp sub-study (1 cup blueberry and placebo groups; *n*=10 per group) participants attended the Cambridge University Hospital CRF for the separate assessment. The [6-6-^2^H_2_] glucose tracer was produced at Guy’s Hospital (QC, Pharmacy Production Unit, London, UK) with commercially labelled D-glucose powder (6,6-D_2_, 99%; Cambridge Isotope Laboratories Inc, Andover, USA). After a standardised evening meal subjects fasted overnight (≥10h). Baseline blood samples were taken, followed by continuous infusion of 0.02mg/kg/min [6,6-^2^H_2_] glucose for 360 min. After 120min (basal/tracer equilibrium phase), insulin (Actrapid, NovoNordisk, Crawley, UK; in 4% autologous blood/saline) was infused at a ‘low’ dose (0.3mU/ kg/min; 2.5 ml/hr for 120min) and then a ‘high’ dose (1.5mU/kg/min; 12.5ml/hr for 120min) with maintenance of plasma glucose level at 0.5mM below baseline by continuous titrated infusion of 20% dextrose (spiked with 8mg (for the low dose infusion) and 10mg (for the high dose) [6,6-^2^H_2_]glucose/g dextrose. Plasma glucose was measured *per* 5 minutes using the glucose oxidase method (YSI glucose analyser, MODEL 2300, Yellow Springs Instruments, USA) until the end of the 2-step study protocol. ‘Steady state’ periods were identified as the last 30min of each phase, during which blood samples were taken *per* 10min for measurement of plasma glucose enrichment, insulin and non-esterified fatty acids (NEFA). After centrifugation (5min; 4000rpm, 4°C; Thermo Heraeus Fresco 17, Waltham, USA) samples were aliquoted and stored at -80°C until later analysis. Glucose tracer enrichment was determined by gas chromatography-mass spectrometry (GC-MS); 5971A MSD (Agilent Technologies, Wokingham, Berks, UK) and expressed as tracer to tracee ratio. Rates of glucose appearance (Ra) and disposal (Rd) were calculated using the non-steady state Steele model with modification to incorporate the use of a stable isotope (2, 3); Optimal Segments Technique Analysis was applied to smooth glucose concentration and enrichment time courses (4). Metabolic clearance rate (MCR) was calculated as glucose Rd/plasma glucose concentration. Plasma non-esterified fatty acids were assessed using a commercially available enzymatic colorimetric assay (Roche, kit 11383175001, Basel, Switzerland) according to manufacturer’s instructions; a CV of 7.3% (NEFA) was reported. Fasting plasma insulin was measured on a Diasorin Liaison® XL automated immunoassay analyser using a one-step chemiluminescence immunoassay (Kit No. 310360; both Diasorin S.p.A, Saluggia, Italy) and international reference standards (WHO International Reference Parameters 66/304). Insulin and NEFA analysis were performed at the Core Biochemical Assay Laboratory (Cambridge University Hospitals, Cambridge, UK); situated within the hospital where the clamp was performed. Due to logistical issues, the ethics committee approved the researchers to compared full dose *versus* placebo dose on 20JAN2014.

*Vascular function assessments*

Brachial artery flow mediated dilatation (FMD) was assessed by ultrasound (Philips iE33; 11-3MHz linear transducer, Philips, Surrey, UK) with image acquisition triggered by 3-lead ECG gating (Vascular Imager software; Medical Imaging Applications LLC, Coralville, USA). A standardized protocol was followed; 1min baseline, followed by reactive hyperemia (induced via 220*mmHg* sphygmomanometric cuff inflation for 5min), with post-occlusion artery diameter assessed, after rapid cuff deflation, for 5min. FMD image sequences were independently assessed (by PC and LB), using automated edge-detection software (Brachial Analyzer v5; Medical Imaging Applications LLC). Subsequently, mean baseline brachial artery diameter was averaged across all viable frames (pre-occlusion) and maximum diameter was averaged over ≥3 sequential frames at peak diameter (post-occlusion). Percentage FMD (%FMD) was calculated as (diameter_max_−diameter_baseline_) / diameter_baseline_×100. Data was excluded when both researchers independently scored image sequences as ‘failed’. The %FMD recorded was the mean of the independent researcher analysis. BP measurements were taken in triplicate (separated by 3min) using an automated sphygmomanometer with an appropriately sized cuff (Omron 705IT, Omron Healthcare Co., Kyoto, Japan), following 15min of supine rest in a quiet, temperature monitored clinical room (21–24°C). Similarly, Aortic distensibility was assessed via carotid-femoral pulse wave velocity (cfPWV) with transit time automatically calculated (Vicorder, Smart medical, UK) between the initiation of pulse at the carotid and femoral sites. Anatomical distances were measured in duplicate (to the nearest 0.1 cm) by the research staff prior to assessment; including distances between carotid and femoral cuff placement, upper sternal notch and shoulder proximity. Systemic arterial stiffness was assessed via augmentation index (AIx) which measured the wave reflection to arterial pressure waveform (automatically generated by Vicorder software, Smart medical, UK) and this was subsequently standardised to 75bpm, based on calculations which utilized heart rate during AIx data capture. Up to six assessments were made with a target of attaining ≤10% CV for cfPWV and AIx. An identical sequence of assessments were performed at baseline and 6-months.

*Anthocyanin metabolite and data analysis*

Upon receipt, serum and urine were acidified using formic acid (>95% reagent grade, Sigma, Dorset UK); 52.5 µL/mL for serum and 32µL/mL for urine. Pre-acidified serum (100µL) and urine samples (50µL) were then extracted using solid phase extraction (SPE, Strata-X 33u Polymeric Reversed Phase, 60mg/1ml well, Phenomenex, Cheshire, UK) based on previous methods (5) and concentrated to 25µL. Samples were injected onto a PFP HPLC column (2.6µM, 100 x 2.1mm; Phenomenex, Cheshire, UK), at 1μL and a column oven temperature of 37°C. The solvent gradient consisted of acetonitrile and water (0.1% formic acid by volume), using a stepped gradient from 1% to 90% acetonitrile and flow rate between 300 and 450 μl/min over 35mins. The HPLC was coupled to an ESI-MS/MS (SCIEX 3200 series Q-trap MS/MS; SCIEX, Warrington, UK) with source parameters: curtain gas 30 psi, ion source gas-1 35 psi, ion source gas-2 50 psi, ion spray voltage -4000V/+4000V, temperature 700°C. Transition-specific collision cell voltages were used, established independently for each compound using syringe infusion from purchased standards (Sigma, Dorset, UK). Matrix matched standard curves were prepared for quantification ranging from 0-10μM (10 points) for serum and from 0-20μM (11 points) for urine and for each compound 6 consecutive points were chosen for quantification. Identification was based on retention time and 3 to 5 unique transitions and quantified relative to the most abundant transition. Peak areas and concentrations were established using Analyst 1.5 software (AB Sciex), which were then corrected (for the final sample volume) using the internal standard *scopoletin*. Data handling was performed in excel, with the corrected datasets subsequently exported to SAS (SAS v9.3/9.4, SAS Institute Inc., Cary, NC, USA) to generate datasets for the final statistical analysis. Total phenolics were calculated as the sum of all individual compounds with their respective limits of detection (LOD) as minimum value. Analytes having greater than 10% missing data were removed from the dataset and the final statistical analysis was performed on 71 metabolites from serum and 72 metabolites from urine. LOD’s were calculated for each compound as 3x the signal to noise (Supplemental **Table 3)**, inter- and intraplate CVs for the internal standard scopoletin were as follows: serum, interplate %CV of 16.9%, intraplate range in CV of 7.8 to 19.4%; urine, interplate %CV of 14.3%, intraplate range in CV of 7.3 to 17.7%. Regarding average linearity: for serum, 0.992, range (min-max) 0.959-0.998, 68% of the compounds > 0.993; for urine, 0.995, range (min-max) 0.983-0.999, 86% of the compounds > 0.993.

**COMPLETENESS OF DATA ANALYSIS**

In the cfPWV analysis, *n*=5 participants declined to participate in the assessment. During blind review, *n*=10 FMD sequences and *n*=5 nitrite/nitrate analyses were excluded (poor imaging, and data from samples coinciding with an HPLC software error, respectively). The baseline food frequency questionnaire of 6 participants were considered invalid; *n*=2 were incompletely completed, *n*=4 had a ratio of energy intake to estimated energy requirements 2SD outside the sample mean at the relevant time point. Eighty-seven participants reported complete 24 h urine samples collections at both time points and were included in the urine metabolite analysis. One participant in the 1 cup blueberry group commenced BP medication during the study (data excluded from vascular assessments and analysis of related hemodynamic biomarkers).

**SUPPLEMENTAL DISCLOSURES**

The blueberries (freeze dried) and the matched placebo were produced by The United States Highbush Blueberry Council and The National Food laboratory (US) respectively. Insulin clamp studies in Cambridge were supported by the National Institute for Health Research - Cambridge Biomedical Research Centre. The University of Cambridge received salary support for MLE from the National Health Service in the East of England through the Clinical Academic Reserve. AC is a Royal Society Wolfson Research Merit Award Holder. Requests for access to the study protocol will be considered by the corresponding author.

**SUPPLEMENTAL TABLES**

**Supplemental Table 1.** Instructions provided to volunteers regarding food restrictions for 21days prior to, and during, the study.

Please limit the intake of foods in Column A (and foods that contain these items) to the number of portions in Column B. Examples of alternative *non-restricted* foods are shown in Column C.

| **Column A** | **Column B** | **Column C** |
| --- | --- | --- |
| **Foods; portion size** | **Instructions for intake / limit to a frequency of;** | **Alternatives examples** |
| ***Fruits*** | | |
| Blueberry fruit, or foods containing blueberry | AVOID for 21d before and during the 6-month study | e.g. banana, orange, mango, melon, green grapes, peach, pear, nectarine |
| Blackcurrant, blackberry and cranberry, bilberry, black raspberry; *handful* | 1 portion per week (in total, **not** per item) |  |
| Cherry; *handful* |  |  |
| Red / Black / Purple grapes; *handful* |  |  |
| Strawberry, raspberry (red); *80g serving* | 1 portion per week (in total, **not** per item) |  |
| Redcurrant, lingonberries, black olives; *handful* |  |  |
| ***Vegetables*** | | |
| Red cabbage, Aubergine; *medium portion* | 1 portion per week (in total, **not** per item) | e.g. white cabbage, cauliflower, lettuce |
| Radicchio, black beans; medium portion | 2 portions per week (in total, **not** per item) |  |
| ***Miscellaneous*** | | |
| Dark chocolate (e.g. 70% cocoa solids); *portion of 5 chunks* | 2 portions per week | e.g. milk chocolate, white chocolate |
| Oily fish (e.g. salmon, mackerel, fresh tuna, herring); *medium portion* | 2 portions per week (in total, **not** per item) | e.g. white fish, non-oily fish |
| ***Beverages*** | | |
| *Red wine; small glass (125mL) | 1 small glass per week (in total) | e.g. white wine, or other alcoholic drinks such as; lager, cider, bitter, spirits |
| Tea (including herbal), coffee (instant or filter); *medium mug* | 4 cups per day (in total, **not** per item) | Malted drinks (**not** dark chocolate) |
| *Alcohol; *units* | ≤14 units women  ≤21 units men (UK recommendation) | Non-alcoholic drinks, fruit juices (**not** berry juice) |

**Supplemental Table 2.** Instructions provided to volunteers regarding additional food restrictions for 24h prior to each assessment visit.

The following is a list of foods and beverages that were to be avoided (in RED) for 24hours before each of the assessments

| **Foods to avoid for 24hr (*In addition to supplemental Table 1)** | **Alternatives examples** |
| --- | --- |
| **Vegetables, salad and products (e.g. quiche, pizza, casserole) containing:** | |
| **Beetroot, broccoli, cabbage, carrot, cauliflower, celery, cucumber, lettuce, Brussels sprouts, parsley, potato, radish, spinach** | **Onion (excluding red), shallot, green bean, tomato, peas, leek, pumpkin, parsnip, pepper, lentil, sweet corn, green olive** |
| **Other foods: including products (e.g. roll, sandwich) containing the following:** | |
| **Drinks containing caffeine e.g. tea, coffee, hot chocolate, coke, energy drinks (such as RedBull)**  **Bottled water (excluding Buxton mineral water)**  **Drinks / foods containing alcohol**  **Cured and canned meat (e.g. bacon, ham, sausages, corned beef)**  **Smoked fish** | **Milk, Buxton water (provided by the researchers for the 24hr before you attend an assessment visit)**  **Fresh meat (e.g. chicken, turkey, beef, pork, lamb)**  **Fresh fish** |

**Supplemental Table 3.** Compound-specific quality parameters of phenolic acids (nmol/L) in LCMS methods.

| **Compound** | **Mode** | **LOD^1^ Serum** | | **LOD Urine** | |
| --- | --- | --- | --- | --- | --- |
|  |  | **Mean** | **SD** | **Mean** | **SD** |
| 2,3-dihydroxybenzoic acid | (-) | 389 | 304 | 736 | 174 |
| 2,4,6-trihydroxybenzaldehyde | (-) | 95 | 38 | 91 | 32 |
| 2,4-dihydroxybenzaldehyde | (+) | N/A^2^ | - | 3395 | 850 |
| 2,5-dihydroxybenzoic acid | (-) | N/A | - | 180 | 50 |
| 2,6-dihydroxybenzoic acid | (-) | 116 | 41 | N/A | - |
| 2,6-dimethoxybenzoic acid | (-) | 501 | 156 | 597 | 184 |
| 2-hydroxy-4-methoxybenzaldehyde | (+) | 965 | 432 | N/A | - |
| 2-hydroxy-4-methoxybenzoic acid | (-) | 17 | 2 | 20 | 8 |
| 2-hydroxy-6-methoxybenzoic acid | (-) | 63 | 19 | 59 | 11 |
| 2-hydroxybenzoic acid | (-) | 64 | 28 | 86 | 9 |
| 2-hydroxycinnamic acid | (-) | 46 | 32 | 48 | 17 |
| 3,4,5-trihydroxybenzoic acid | (-) | 48 | 13 | 72 | 10 |
| 3,4,5-trihydroxybenzoic acid-methyl ester | (-) | 26 | 15 | 26 | 13 |
| 3,4,5-trimethoxybenzaldehyde | (+) | 50 | 14 | 55 | 13 |
| 3,4,5-trimethoxybenzoic acid-methyl ester | (+) | 309 | 236 | N/A | - |
| 3,4,5-trimethoxyphenylpropionic acid | (-) | 349 | 87 | 594 | 378 |
| 3,4-dihydroxy-5-methoxybenzoic acid | (+) | 490 | 248 | 1208 | 147 |
| 3,4-dihydroxybenzaldehyde | (-) | 55 | 25 | 48 | 20 |
| 3,4-dihydroxybenzoic acid | (-) | 52 | 9 | 57 | 14 |
| 3,4-dihydroxybenzoic acid-methyl ester | (-) | 27 | 18 | 28 | 10 |
| 3,4-dihydroxycinnamic acid | (-) | 262 | 74 | 236 | 117 |
| 3,4-dihydroxyphenylacetic acid | (-) | 405 | 219 | 127 | 73 |
| 3,4-dihydroxyphenylpropionic acid | (-) | 348 | 189 | 506 | 331 |
| 3,4-dimethoxybenzoic acid | (-) | 633 | 302 | 1976 | 794 |
| 3,4-dimethoxybenzoic acid-methyl ester | (+) | 54 | 25 | 47 | 9 |
| 3,4-dimethoxybenzyl alcohol | (+) | 665 | 348 | N/A | - |
| 3,4-dimethoxyphenylacetic acid | (-) | 51 | 15 | 51 | 25 |
| 3,4-methylhippuric acid | (-) | 109 | 87 | 140 | 80 |
| 3,5-dihydroxybenzaldehyde | (-) | 182 | 60 | 206 | 38 |
| 3,5-dihydroxybenzoic acid | (-) | 157 | 32 | 60 | 6 |
| 3,5-dihydroxybenzoic acid-methyl ester | (-) | N/A | - | 160 | 71 |
| 3,5-dihydroxybenzylalcohol | (-) | 98 | 21 | 169 | 63 |
| 3,5-dihydroxyphenylproponoic acid | (-) | 171 | 83 | 94 | 19 |
| 3,5-dimethoxybenzaldehyde | (+) | 336 | 226 | N/A | - |
| 3,5-dimethoxybenzoic acid-methyl ester | (+) | 59 | 35 | 59 | 25 |
| 3-hydroxy-4-methoxybenzaldehyde | (-) | 1781 | 1867 | N/A | - |
| 3-hydroxy-4-methoxybenzoic acid | (+) | 1117 | 482 | 141 | 43 |
| 3-hydroxy-4-methoxycinnamic acid | (-) | 170 | 62 | 198 | 53 |
| 3-hydroxy-4-methoxyphenylacetic acid | (-) | 719 | 209 | 840 | 105 |
| 3-hydroxybenzaldehyde | (-) | 133 | 34 | 163 | 34 |
| 3-hydroxybenzoic acid | (-) | 152 | 31 | 151 | 43 |
| 3-hydroxybenzoic acid-4-glucuronide | (-) | 12 | 3 | 20 | 7 |
| 3-hydroxybenzoic acid-4-sulfate & 4-hydroxybenzoic acid-3-sulfate* | (-) | 7 | 5 | 13 | 7 |
| 3-hydroxybenzoic acid-methyl ester | (-) | N/A | - | 1292 | 426 |
| 3-hydroxyhippuric acid | (-) | 125 | 100 | 13 | 11 |
| 3-hydroxyphenylpropionic acid | (-) | 99 | 29 | 95 | 20 |
| 3-methoxybenzoic acid | (+) | N/A | - | 431 | 412 |
| 3-methoxybenzoic acid-4-glucuronide | (-) | 21 | 14 | 24 | 7 |
| 3-methoxybenzoic acid-4-sulfate & 4-methoxybenzoic acid-3-sulfate* | (-) | 14 | 9 | 12 | 5 |
| 3-methoxybenzoic acid-methyl ester & 4-methoxybenzoic acid-methyl ester* | (+) | 237 | 86 | 403 | 63 |
| 3-methoxyphenylpropionic acid | (-) | N/A | - | 1958 | 741 |
| 4-hydroxy-2-methoxybenzaldehyde | (+) | N/A | - | 318 | 92 |
| 4-hydroxy-3,5-dimethoxybenzoic acid | (+) | 80 | 45 | 68 | 14 |
| 4-hydroxy-3,5-dimethoxycinnamic acid | (-) | 15 | 4 | 14 | 2 |
| 4-hydroxy-3,5-dimethoxyphenylacetic acid | (-) | N/A | - | 595 | 188 |
| 4-hydroxy-3-methoxybenzoic acid | (-) | 153 | 87 | 135 | 57 |
| 4-hydroxy-3-methoxycinnamic acid | (-) | 49 | 12 | 35 | 11 |
| 4-hydroxy-3-methoxyphenylacetic acid | (-) | 1020 | 322 | 1226 | 464 |
| 4-hydroxy-3-methoxyphenylpropionic acid | (-) | 42 | 7 | 74 | 80 |
| 4-hydroxybenzaldehyde | (-) | 76 | 63 | 49 | 35 |
| 4-hydroxybenzoic acid | (-) | 78 | 33 | 72 | 17 |
| 4-hydroxybenzoic acid-3-glucuronide | (-) | 11 | 8 | 21 | 10 |
| 4-hydroxybenzoic acid-methyl ester | (-) | 18 | 5 | 23 | 8 |
| 4-hydroxybenzyl alcohol | (-) | N/A | - | 135 | 60 |
| 4-hydroxycinnamic acid | (-) | 64 | 48 | 58 | 16 |
| 4-hydroxyhippuric acid | (-) | 11 | 4 | 4 | 2 |
| 4-hydroxyphenylacetic acid | (-) | 1007 | 305 | N/A | - |
| 4-hydroxyphenylpropionic acid | (-) | 428 | 203 | 1447 | 471 |
| 4-methoxybenzoic acid | (+) | 250 | 46 | N/A | - |
| 4-methoxybenzoic acid-3-glucuronide | (-) | 27 | 4 | 31 | 6 |
| 4-methoxyphenylpropionic acid | (+) | 1213 | 963 | 1196 | 304 |
| Benzoic acid^3^ | (-) | N/A | - | 14 | 0 |
| benzoic acid-4-glucuronide | (-) | 11 | 4 | 25 | 9 |
| Benzoic acid-4-sulfate | (-) | 25 | 6 | 11 | 9 |
| Benzoylglutamic acid | (-) | 23 | 7 | 24 | 8 |
| Chlorogenic acid | (-) | 47 | 24 | 34 | 13 |
| Cyanidin-3-glucoside | (+) | 20 | 15 | 33 | 9 |
| Hippuric acid^3^ | (-) | 85 | 108 | 8 | 0 |
| Rosmarinic acid | (-) | 8 | 3 | 7 | 2 |
| trans-3-hydroxycinnamic acid | (-) | 79 | 56 | 75 | 24 |

^1^Limits of Detection (LOD) represent the average over 7 plates calculated as 3 times the signal to noise.

^2^ N/A, concentration data not available for this compound

^3^For Benzoic acid and Hippuric acid in urine, concentrations were calculated using an external standard curve extending to 2000μM, at which point the LOD was established. As the LOD is not calculated over 7 plates, the SDs of these LODs in urine are 0.

* Where two compounds are reported together, they could not be sufficiently resolved.

**Supplemental Table 4.** Change in peripheral, hepatic and adipose insulin sensitivity in a 2-step hyperinsulinemic euglycemic clamp sub-study (1 US cup blueberries *versus* placebo).^1-3^

|  | Placebo (n=8) | | | 1 cup (n=8) | | |  |
| --- | --- | --- | --- | --- | --- | --- | --- |
|  | Before | After | ∆ 0 to 6M | Before | After | ∆ 0 to 6M | P= |
| Fasting phase |  |  |  |  |  |  |  |
| HOMA-IR | 2.2 (1.8, 2.7) | 2.2 (1.7, 2.6) | -0.03 (-0.63, 0.57) | 2.8 (2.3, 3.3) | 1.7 (1.2, 2.2) | -1.08 (-1.72, -0.44) | 0.02 |
| NEFA (µmol/L) | 327 (255, 398) | 367 (295, 438) | 40.2 (-60.0, 140) | 347 (271, 424) | 387 (311, 464) | 40.1 (-67.1, 147) | 1.00 |
| Low insulin step phase | | | | | | | |
| Insulin (pmol/L) | 175 (164, 187) | 179 (168, 191) | 3.9 (-12.3, 20.1) | 176 (165, 188) | 181 (170, 193) | 5.1 (-11.2, 21.3) | 0.92 |
| Ra (µmol/kgBW/min) | 3.4 (2.8, 3.9) | 3.0 (2.4, 3.6) | -0.37 (-1.16, 0.41) | 3.3 (2.8, 3.9) | 3.0 (2.4, 3.6) | -0.34 (-1.12, 0.44) | 0.95 |
| MCR (ml/kgBW/min) | 2.7 (2.4, 3.0) | 2.6 (2.3, 2.9) | -0.05 (-0.46, 0.37) | 2.6 (2.4, 2.9) | 2.3 (2.0, 2.5) | -0.39 (-0.81, 0.02) | 0.25 |
| NEFA (µmol/L) | 76.9 (48.4, 105) | 90.0 (61.4, 118) | 13.1 (-25.2, 51.3) | 79.0 (50.5, 107) | 74.9 (46.4, 103) | -4.04 (-42.3, 34.2) | 0.54 |
| NEFA % suppression | 81.6 (74.0, 89.2) | 79.8 (72.2, 87.4) | -1.79 (-12.3, 8.7) | 79.1 (70.9, 87.3) | 81.7 (73.6, 89.9) | 2.6 (-8.62, 13.9) | 0.57 |
| High insulin step phase | | | | | | | |
| Insulin (pmol/L) | 916 (849, 982) | 917 (851, 984) | 1.8 (-92.4, 96.0) | 910 (843, 977) | 923 (856, 989) | 12.5 (-81.7, 107) | 0.87 |
| Rd (mg/kgBW/min) | 47.4 (42.8, 52.0) | 45.0 (40.4, 49.6) | -2.40 (-8.84, 4.0) | 45.9 (41.3, 50.6) | 41.6 (36.9, 46.2) | -4.35 (-10.8, 2.1) | 0.67 |
| MCR (ml/kgBW/min) | 9.5 (8.5, 10.5) | 8.3 (7.4, 9.3) | -1.20 (-2.54, 0.14) | 9.1 (8.2, 10.1) | 8.1 (7.1, 9.1) | -1.04 (-2.38, 0.30) | 0.87 |

^1^

Ra indicates, rate of appearance (hepatic glucose production); Rd, rate of disposal (peripheral glucose uptake); MCR, metabolic clearance rate (MCR = glucose Rd / plasma glucose concentration); NEFA, non-esterified fatty acid (indicator of adipose tissue lipolysis).

^1^Values are mean (95% CI) adjusted for baseline values only; adjusting for age, sex, change in BMI and baseline statin use did not fundamentally change the results. Cup, relates to the equivalent number of US cups of fresh blueberries. P= p values for the time x treatment interaction using a linear mixed-effect model. *n*=16 participants, of which *n*=5 female (*n*=3 placebo, *n*=2, in the 1 US cup group).

^2^ Percent suppression of NEFA from fasting = (fasting NEFA – clamped NEFA) / (fasting NEFA) * 100. Low and high, refer to the steady state period of the low dose insulin and high dose insulin phases of the clamp assessment, respectively.

^3^ Data exclusions: n=1 missing data for fasting insulin, HOMA-IR, NEFA (1 cup group).

**Supplemental Table 5.** Change in aortic distensibility (cfPWV) and vascular function biomarkers from baseline to six months by intervention group.^1,2^

|  | Placebo (n=39) | | | 1/2 cup (n=39) | | | 1 cup (n=36) | | |  |
| --- | --- | --- | --- | --- | --- | --- | --- | --- | --- | --- |
|  | Before | After | ∆ 0 to 6M | Before | After | ∆ 0 to 6M | Before | After | ∆ 0 to 6M | P= |
| cfPWV (m/s)^2^ | 10.9  (10.7, 11.2) | 10.7  (10.5, 10.9) | -0.27  (-0.57, 0.03) | 10.8  (10.6, 11.0) | 10.5  (10.3, 10.7) | -0.24  (-0.51, 0.04) | 10.8  (10.6, 11.0) | 10.9  (10.6, 11.1) | 0.05  (-0.28, 0.38) | 0.31 |
| RXNO (nM)^2^ | 14.8  (13.5, 16.1) | 12.4  (11.2, 13.7) | -2.40  (-3.86, -0.94) | 14.5  (13.1, 15.9) | 14.0  (12.6, 15.4) | -0.49  (-2.25, 1.3) | 14.3  (12.8, 15.7) | 13.7  (12.3, 15.1) | -0.59  (-2.37, 1.2) | 0.15 |
| Nitrite (uM)^2^ | 1.1  (0.95, 1.2) | 1.0  (0.93, 1.1) | -0.03  (-0.09, 0.03) | 1.0  (0.93, 1.2) | 0.96  (0.85, 1.1) | -0.09  (-0.18, 0.01) | 1.1  (0.97, 1.2) | 1.1  (1.0, 1.3) | 0.04  (-0.07, 0.15) | 0.21 |
| Nitrate (uM)^2^ | 27.5  (24.5, 30.5) | 32.1  (29.0, 35.1) | 4.6  (1.2, 8.0) | 28.0  (24.9, 31.0) | 31.3  (28.2, 34.5) | 3.4  (-0.38, 7.1) | 28.9  (26.0, 31.7) | 29.1  (26.2, 32.0) | 0.22  (-2.60, 3.0) | 0.13 |
| Total Free Thiols (µM)^2^ | 281  (275, 287) | 284  (278, 290) | 3.1  (-5.48, 11.6) | 277  (271, 284) | 280  (273, 286) | 2.0  (-6.66, 10.7) | 280  (273, 287) | 287  (281, 294) | 7.1  (-1.77, 15.9) | 0.71 |

cfPWV indicates carotid-femoral pulse wave velocity; and RXNO, S-nitrosothiols + other nitroso species.

^1^Values are mean (95% CI) adjusted for baseline values, age, change in BMI, sex, baseline anti-hypertensive and statin use. Cup, relates to the equivalent number of US cups of fresh blueberries. *P=* p values for the time x treatment interaction calculated using a linear mixed-effect model; letters indicate significant differences between groups a= 1 cup vs. placebo, b= ½ cup vs. placebo and c= 1 cup vs. ½ cup.

^2^ Data exclusions: cfPWV, *n*=16 missing data; a maximum of *n*=4 outliers were identified. *n*=1 excluded from all analyses due to change in BP medication during study.

**Supplemental Table 6.** Extended overview of the change in lipid, lipoprotein and apolipoprotein levels, from baseline to six months, by statin non-users (n=71) grouped by intervention.^1,2^

|  | Placebo (n=24) | | | ½ cup (n=23) | | | 1 cup (n=24) | | |  |
| --- | --- | --- | --- | --- | --- | --- | --- | --- | --- | --- |
|  | Before | After | ∆ 0 to 6M | Before | After | ∆ 0 to 6M | Before | After | ∆ 0 to 6M | P= |
| HDL-C (mmol/L) | 1.1  (1.1, 1.2) | 1.1  (1.1, 1.1) | -0.03  (-0.08, 0.02) | 1.1  (1.1, 1.2) | 1.2  (1.1, 1.2) | 0.02  (-0.03, 0.06) | 1.1  (1.1, 1.2) | 1.2  (1.2, 1.2) | 0.05  (0.01, 0.10)^a^ | 0.05 |
| HDL-P, n (x10-6) | 7.48  (7.33, 7.64) | 7.37  (7.22, 7.53) | -0.11  (-0.33, 0.10) | 7.47  (7.32, 7.62) | 7.63  (7.48, 7.78) | 0.16  (-0.05, 0.37) | 7.47  (7.32, 7.62) | 7.84  (7.68, 7.99) | 0.37  (0.16, 0.58)a | 0.01 |
| Apo-A1 (g/L) | 1.33  (1.31, 1.35) | 1.32  (1.30, 1.34) | -0.01  (-0.04, 0.02) | 1.33  (1.31, 1.35) | 1.36  (1.34, 1.38) | 0.03  (0.00, 0.06)b | 1.33  (1.31, 1.35) | 1.37  (1.35, 1.39) | 0.04  (0.01, 0.07)a | 0.03 |
| Chol: HDL-C | 5.3  (5.1, 5.4) | 5.2  (5.1, 5.4) | -0.04  (-0.27, 0.19) | 5.3  (5.1, 5.4) | 5.4  (5.2, 5.6) | 0.13  (-0.09, 0.35) | 5.3  (5.1, 5.4) | 5.1  (5.0, 5.3) | -0.12  (-0.34, 0.10) | 0.28 |
| TG (mmol/L) | 1.9  (1.8, 2.1) | 1.9  (1.7, 2.0) | -0.06  (-0.27, 0.14) | 1.9  (1.8, 2.1) | 2.2  (2.1, 2.4) | 0.31  (0.10, 0.51)^b^ | 1.9  (1.8, 2.1) | 2.0  (1.8, 2.1) | 0.05  (-0.15, 0.25) | 0.04 |
| LDL-P, n (x10-6) | 0.48  (0.46, 0.49) | 0.48  (0.46, 0.49) | 0.00  (-0.02, 0.02) | 0.48  (0.46, 0.49) | 0.49  (0.47, 0.51) | 0.01  (-0.01, 0.03) | 0.48  (0.46, 0.49) | 0.49  (0.48, 0.51) | 0.02  (-0.01, 0.04) | 0.49 |
| ApoB (g/L) | 0.96  (0.93, 0.98) | 0.95  (0.92, 0.98) | -0.01  (-0.05, 0.03) | 0.96  (0.93, 0.98) | 1.00  (0.97, 1.03) | 0.04  (0.01, 0.08) | 0.95  (0.93, 0.98) | 0.97  (0.95, 1.00) | 0.02  (-0.02, 0.06) | 0.18 |

HDL-P, n, indicates high density lipoprotein particle number; Apo-A1, apolipoprotein A1; TG, triglycerides; LDL-P, n, low density lipoprotein particle number; and ApoB, apolipoprotein B.

^1^Values are mean (95% CI) adjusted for baseline values, age, change in weight, sex. Cup, relates to the equivalent number of US cups of fresh blueberries. *P=* p values for the time x treatment interaction calculated using a linear mixed-effect model; letters indicate significant differences between groups a= 1 cup vs. placebo, b= ½ cup vs. placebo and c= 1 cup vs. ½ cup.

^2^ Data exclusions: *n*=1 outlier (1 cup group) for Apo-A1, LDL-P and ApoB analysis.

**SUPPLEMENTAL REFERNCES**

1. IPAQ core group.

2. Steele R. Influences of glucose loading and of injected insulin on hepatic glucose output. Annals of the New York Academy of Sciences 1959;82:420-30.

3. Finegood DT, Bergman RN, Vranic M. Estimation of endogenous glucose production during hyperinsulinemic-euglycemic glucose clamps. Comparison of unlabeled and labeled exogenous glucose infusates. Diabetes 1987;36(8):914-24.

4. Finegood DT, Bergman RN. Optimal segments: a method for smoothing tracer data to calculate metabolic fluxes. The American journal of physiology 1983;244(5):E472-9.

5. de Ferrars RM, Czank C, Saha S, Needs PW, Zhang Q, Raheem KS, Botting NP, Kroon PA, Kay CD. Methods for isolating, identifying, and quantifying anthocyanin metabolites in clinical samples. Analytical chemistry 2014;86(20):10052-8. doi: 10.1021/ac500565a.
